# Ochratoxin A in Ruminants–A Review on Its Degradation by Gut Microbes and Effects on Animals

**DOI:** 10.3390/toxins204809

**Published:** 2010-04-21

**Authors:** Muhammad Mobashar, Jürgen Hummel, Ralf Blank, Karl-Heinz Südekum

**Affiliations:** 1 Institute of Animal Science, University of Bonn, Endenicher Allee 15, 53115 Bonn, Germany; Email: mmob@itw.uni-bonn.de (M.M.); jhum@itw.uni-bonn.de (J.H.); 2 Institute of Animal Nutrition and Physiology, Christian-Albrechts-University Kiel, 24098 Kiel, Germany; Email: blank@aninut.uni-kiel.de (R.B.)

**Keywords:** OTA, mycotoxin, enzymatic degradation, diet, protozoa, carry over, milk

## Abstract

Ruminants are much less sensitive to ochratoxin A (OTA) than non-ruminants. The ruminal microbes, with protozoa being a central group, degrade the mycotoxin extensively, with disappearance half lives of 0.6–3.8 h. However, in some studies OTA was detected systemically when using sensitive analytical methods, probably due to some rumen bypass at proportions of estimated 2–6.5% of dosage (maximum 10%). High concentrate proportions and high feeding levels are dietary factors promoting the likeliness of systemic occurrence due to factors like shifts in microbial population and higher contamination potential. Among risk scenarios for ruminants, chronic intoxication represents the most relevant.

## 1. Introduction

Ochratoxin A (OTA) is a secondary metabolite of some toxigenic storage fungal species of the genera *Aspergillus* and *Penicillium* [1,2]. It has a widespread occurrence in foods and feedstuffs [3], e.g., in Canada, Denmark, Germany, Sweden and United Kingdom [4]. In tropical and subtropical areas OTA is mainly produced by *Aspergillus* species (mainly *A. ochraceus*) whereas in temperate regions *Penicillium* species are of great importance, especially *P. verrucosum* [1]. Several forms of ochratoxin occur, showing varying degrees of toxicity. OTA has been shown to be dominantly nephrotoxic; however, hepatotoxic, teratogenic and carcinogenic effects are also discussed [5].

Negative effects of OTA can be considered on different levels of the food chain. While the most alarming would be any relevant contamination of animal products, it needs to be stated clearly that OTA level in human diets is low in general [6], and that the contribution of animal products to total OTA intake does not exceed 3–10% in human nutrition [7]. More significant proportions come from grains (54%), red wine (15%) or coffee (12%) [6]. As desirable as further minimisation of any residual risk of OTA for the human food chain may be, the major perspective of studies on OTA in animal feeds is the reduction of negative effects on animals and their welfare and the evaluation of the level of OTA contaminated material that can be used in rations without negative effects.

It is a general experience in animal production that toxic effects of OTA are much more pronounced for non-ruminants like pigs than for ruminants. The evolution of forestomach fermentation has been regarded to have been triggered by the potential for detoxification of plant toxins [8], and the system works effectively for toxins like OTA, too: There is convincing evidence that gut microbes play the key role in OTA degradation and detoxification in ruminants. OTA is a complex compound consisting of ochratoxin α (OTα) linked through a 7-carboxy group to L-β-phenylalanine by an amide bond. Hydrolysis of this bond by microorganisms within the gastrointestinal tract leads to the far less or even non-toxic OTα and phenylalanine, which is often considered as the principal means of detoxification of OTA in ruminants [9].

In theory, the tolerance of ruminants to the toxic effects of OTA gives them some potential as users of contaminated feeds. However, recently some doubts and discrepancies have been raised to what extent and in which amounts OTA is sufficiently detoxified by ruminants under all feeding regimes/circumstances [10,11]. It is not clear to what extent particular microbial groups like protozoa contribute to OTA degradation and factors like the degree of contamination of typical feedstuffs need to be quantified.

Several reviews on different aspects of OTA have been published including overviews on general aspects [12,13,14,15,16], on OTA as a storage mycotoxin [17], on OTA and its mode of action [18,19,20], on carcinogenic potential of OTA [21], on general risk assessment of OTA [22] or on decontamination [23,24].

This review is intended to contribute to a better understanding of the significance of OTA for ruminants. Since OTA contamination of diets can be expected to be mostly below a level relevant for toxic effects in ruminants, the potential of eventual aberrations from this rule will be pointed out and quantified. This appears warranted given the toxic potential of OTA. After evaluating the contamination of ruminant diets, a closer, quantitative look is depicted at factors contributing to OTA metabolism in ruminants, starting with microbial degradation of OTA. This is followed by an evaluation of systemic occurrence of OTA in ruminants and a summary of the evidence for the occurrence in ruminant products.

## 2. Occurrence of Ochratoxin A in Ruminant Feeds

Given the detoxification potential of ruminants and the generally low indication for OTA occurrence in forages, it is not surprising that OTA contamination is not at the centre of mycotoxin concerns in ruminant feeds [25]. However, some OTA contamination of diets can not be excluded given the variety of feeds offered to ruminants. While it can sometimes occur at concentrations high enough to cause major losses in health and performance of monogastrics, a more likely scenario for ruminants is to find a lower level of OTA occurrence in diets and only subclinical disorders related with OTA.

### 2.1. Concentrates

OTA has been shown to occur in various grains and other plant products throughout the world [26,27,28,29,30,31,32]. It is generally observed that concentrates are more prone to the growth of OTA producing moulds, especially in cereal feeds such as maize, barley, oats, wheat or rye and in mixed feed [33]. OTA seems to occur more frequently and with a tendency for higher contents in mixed feeds compared to cereal grains, although it originates dominantly from grains in this feed group, too [34,35], cited in [23].

In a study on natural occurrence of OTA in feed concentrates from 13 countries including Canada and the United States until 1983, the percentage of feed that was contaminated ranged from 1 to 30% [1]. In Canada and the United States the contamination range was from 1 to 14.2%. OTA contamination level in animal feeds ranged by country and commodity has been highest in Northern Europe and North America according to the World Health Organization [33]. Data of the WHO show that the countries with the highest frequency of OTA contaminated samples of animal feed were Denmark (57.6%), Canada (56.3%), and Yugoslavia (25.7%). OTA contaminations detected in each of these countries were in the range 30–27,000 µg/kg, 28–27,500 µg/kg, and 45–5,125 µg/kg, respectively. 

A summary of several studies evaluating OTA levels in concentrates (particularly in a variety of cereals in Europe) is shown in Table 1, with concentration levels varying considerably. Incidence of OTA in concentrates is higher in ecological farms than in conventional farms, as has been proven in rye, wheat and barley [36], potentially due to no use of fungicides.

A survey on occurrence of mycotoxins in wheat and maize from western Romania found other mycotoxins as the major contaminants in wheat (n = 25) and maize (n = 30) [45]. OTA was only found in wheat in concentrations of 37 µg/kg in one sample. A study focusing on Brasilian dairy feeds found incidencies of OTA positive samples of 25% for finished cow´s feed, 31% for maize, 22% for barley rootlets and 45% for brewers grains [46]. In dairy feeds used in 5 Sudanese farms, OTA contamination for one manufactured ration (0.22–0.61 μg/kg) and feeds like crushed sorghum (0.33–1.58 μg/kg), sunflower cake (1.59 μg/kg), wheat bran (0–0.43 μg/kg) and groundnut cake (0–0.31 μg/kg) was found [47]. Only one of five farms was tested negative for OTA. 

In a survey on Dutch dairy feeds, no OTA was found in compound feed, ensiled by-products or feed commodities [48]. For a variety of South-African feeds also no OTA was detected in the dairy relevant feeds wheat bran, sorghum brewers grains and a compound feed [49].

**Table 1:** Occurrence of ochratoxin A (OTA) in concentrates; table modified from [15].

Country and year	Feed	n	Positive samples	OTA content (µg/kg)	Ref.
Poland (1975–1979)	Barley	137	19 (14%)	2–200	[37]
	Wheat	125	15 (12%)	5–100	[37]
	Rye	83	15 (18%)	4–200	[37]
Denmark (1986–1992)	Wheat	520	165 (32%)	0.05–51	[4]
	Rye	616	256 (42%)	0.05–121	[4]
	Oats	92	40 (43%)	0.05–6	[4]
	Barley	61	17 (28%)	0.05–14	[4]
United States (1999)	Wheat	383	56 (15%)	0.03–31	[38]
	Barley	103	11(10%)	0.1–17	[38]
United Kingdom (1992)	Wheat	50	10 (22%)	1–02	[39]
	Barley	45	12 (26%)	1–20	[39]
Canada (1981–1983)	Wheat	440	5 (<1%)	10–51	[40]
Germany (1991–1993)	Cereals	514	10 (2%)	3–60	[41]
Germany (1982–1987)	Barley	68	10 (15%)	0.1–206	[26]
	Oats	93	12 (135%)	0.1–58	[26]
	Wheat	64	8 (13%)	0.1–137	[26]
	Maize	40	3 (8%)	1.7–82	[26]
Netherlands (1988–1989)	Cereal grains	44	2 (25%)	6–120	[42]
	Legumes	35	10 (28%)	2–37	[42]
Egypt (1995)	Maize	54	8 (14%)	4800	[43]
	Soybean	17	3 (17%)	1600	[43]
	Wheat	26	2 (8%)	800	[43]
Germany (1982–1987)	Mixed feed	630	89 (14%)	0.2–12	[26]
India (1985–1987)	Cattle cake	143	6 (4%)	n.g.	[44]

n = number of samples; n.g. = not given.

### 2.2. Forages

While occurrence of OTA is primarily known and well established for concentrates, a review on OTA in ruminants should also consider any eventual occurrence of OTA in forages, as they normally represent the major component of ruminant diets. As a mycotoxin produced by typical storage fungi, OTA can not be expected to be present in fresh forages like fresh grass or whole-crop maize. While in silages some mould growth can occur under unfavourable aerobic conditions and OTA contaminations *via* additives can not be excluded completely, OTA presence or mould growth in silages is unlikely as long as anaerobic conditions are maintained. In consequence, most studies investigating the occurrence of OTA in silage gave no evidence for a significant occurrence: Some did not mention OTA as relevant in forages in general [50], others explicitly found no OTA in a large sample of Dutch maize, grass and wheat silages [48,51]; in maize silage also no significant amounts of OTA were found [52,53,54,55]. The minor OTA contents detected in one study [52] are not quoted as positive samples by the author herself since the concentrations were within the range of detection limit.

However, individual contributions report on the occurrence of OTA in silages: values of 20–70 μg/kg were given for maize silage [56], in another sample of 10 maize silages suspected to contain mycotoxins, OTA was found in all samples (mean concentration 17 μg/kg; highest content 37 μg/kg) [57] and also another study reported on the occurrence of OTA in maize silage [58]. If dried forage becomes damp and fungal growth occurs, field species of fungi are likely to be gradually replaced by storage fungi such as species of *Penicillium* and *Aspergillus.* The rate at which this happens depends on the storage conditions and management practices. Insufficient drying, condensation, leakage of rain water or insect infestation can lead to further mould growth and heating [59]. The growth of storage fungi on hay can not be excluded therefore. However, the presence of *Aspergillus* or *Penicillium* alone should not be quoted as direct evidence for the presence of OTA [60]. Therefore reports on *A. ochraceus* in different forages [61] or on *P. verrucosum* as a mould in silages and dried forages [62] should prompt further investigations on OTA presence in these forages rather than to be taken as an approval of the existence of OTA in these samples.

Concerning dried forages, no evidence for the occurrence of OTA in Dutch dried forages used on dairy farms was found [51]. A study on horse feeds also found no OTA in Irish and Canadian hays (n = 15) and haylages (n = 34) [63]. OTA was found only in one of 201 straw samples [64]. In contrast, *A. ochraceus* and *P. verrucosum* were described to produce OTA at a high incidence in straw (60%) and hay (48%) [43], with astonishingly high concentrations of 8,000 (straw) and 2,800 μg/kg (hay). *Penicillium* species have a tendency to produce OTA in forages in all seasons. In a Yugoslavian study, different feed samples including forages like hay, dried lucerne and fresh lucerne were found to be contaminated with moulds at a high incidence (up to 95–100% in one year) throughout three years [58]. The concentration of the toxin varied from traces to 400 µg/kg. 

Information cited from different sources depicted that the incidence of OTA contamination is highest in concentrates, however it may also occur in forages. Since non-ruminant animals generally are fed on concentrate-only diets, a lower daily intake of OTA can be presumed for a ruminant compared to a non-ruminant animal. However, when high-concentrate diets or contaminated forages are used, it seems that considerable concentrations of OTA can occur in ruminant diets, too.

## 3. Degradation of Ochratoxin A in Ruminants

Two aspects need to be considered when evaluating the risk potential of OTA for ruminants: degradation of OTA to less toxic OTα and disappearance rate of OTA and metabolites from the animal and its products. In an attempt to summarize the evidence to date, at first principles and particularities of the degradation by the rumen microbial population are reviewed. In the following, studies dealing with systemic occurrence, excretion and the occurrence in animal products from ruminants are summarized.

Different approaches to minimize the toxic effects of OTA contaminated feeds exist, and they may not necessarily include enzymatic destruction of the molecule, see e.g., [23,65,66]. On the level of feeds, adsorption of the substance, heat-dependant degradation, gamma radiation or chemical treatments all have been proposed [24]. 

Enzymatic degradation of a substance by microbes or isolated enzymes is a further option often mentioned in the context of OTA detoxification of food or feedstuffs [67], well-known to be present in the animal from classic studies [68,69]. While other mechanisms like simply non-absorption from the gut, or low overall systemic receptiveness to a toxic substance can influence the reaction to any toxin, degradation by microbes explains much of the resistance of ruminants against OTA.

### 3.1. Principle of enzymatic OTA degradation

Basically, enzymatic degradation of OTA means cleavage of the amide bond into non-toxic phenylalanine and OTα [9,68,69,70,71,72,73]. Proteolytic enzymes are the most likely to develop significant OTA degrading activity. Based on the chemical structure of the OTA molecule one can speculate about the kind of proteolytic enzymes developing the highest degrading activity, and some *in vitro* studies given below have investigated the potential of OTA degradation of particular enzymes. Carboxypeptidases could be considered to be particularly effective: The enzymes cleave amide bonds at the carboxy-terminal end of a peptide, and carboxypeptidase A shows some preference for aromatic amino acids like phenylalanine as the terminal amino acid [74]. Both characteristics appear somewhat reflected in the molecule structure of OTA. In fact, historically carboxypeptidase A was the first enzyme shown to be effective in OTA degradation [75,76,77], and is still used as a standard for activity of OTA degrading enzymes [67]. 

Among other proteolytic enzymes, chymotrypsin showed less and trypsin no OTA degrading activity [75]. Protease A and pancreatin (with protease, lipase and amylase activity) have also been shown to be effective in OTA degradation to some degree [67]. Even a lipase of *A. niger* was described to have relevant OTA degrading activity [78]. Lipases have been shown to have some proteolytic activity, too [79].

Given some effectiveness of chymotrypsin or pancreatin in OTA degradation, one could speculate to what extent degradation is performed by animal enzymes. While results on excretion of OTα in urine of non-ruminants and ruminant calves indicate the occurrence of some OTA cleavage by animal enzymes at least, empiric evidence of the high susceptibility of non-ruminant animals to OTA toxicity implies that degradation by proteolytic enzymes of the small intestine and pancreas has insufficient capacity to degrade OTA adequately. Nevertheless, the most important point is that in non-ruminant animals the enzymes start to degrade OTA only shortly before the proximal jejunum, shown to be the major site of OTA absorption in the rat [80]. 

In contrast to this situation, in foregut-fermenters like ruminants, the molecule has already passed the major site of OTA degradation, the forestomach, before arriving at the major ochratoxin absorption site, the small intestine.

### 3.2. Degradation by different microbial populations

Inhibitory effects of OTA were present for gram-positive bacteria at a pH lower than 7 [81]. However, considerable OTA degrading capacity has been described for different microbial taxa (bacteria: *Acinetobacter calcoaceticus* [82], *Lactobacillus acidophilus* and *Bifidobacterium animalis* [83]; fungi: *Phaffia rhodozyma* [84], *Rhizopus* spp. [85], *Aspergillus* spp. [86,87,88,89], *Pleurotus ostreatus* [90] and *Trichosporon* [91]). Among microbial populations involved in food production, especially those involved in beer brewing [92,93,94] or yoghurt production (e.g., *Streptococcus salivarius*, *Lactobacillus delbruecki* [95]) have been shown to degrade OTA to some extent.

So while obviously a variety of microbes have OTA degrading capacity, gut microbial populations are probably the most well-known and among the most effective. Such populations are those found in the rat caecum [69,96], in human faeces [97], in the pig gut [98] and, most prominently, in the rumen [68,69,99]. As already mentioned, microbial degradation in non-ruminants like rats, pigs or humans will basically take place in the large intestine and therefore after having passed through the major absorption site. As effective as microbial degradation at this site may be, it is of insignificant relevance for the animal regarding OTA detoxification.

### 3.3. Degradation by rumen microbes

#### 3.3.1. OTA degradation by the rumen microbial population

Two lines of evidence from empiric studies have shown that the large capacity of rumen microbes for OTA degradation is closely related to the insensitivity of ruminants to the toxin: Indirectly by showing that non-ruminating calves (without or with very limited functional rumen microbes) are far more affected by negative effects of orally introduced OTA than ruminating calves (with a functional rumen microbial population) [9], and directly by showing the quantitative transformation of OTA to OTα by rumen microbes *in vitro* or *in vivo* [68,69,99]. In contrast to extensive degradation by inoculum from the three forestomach compartments (rumen, reticulum, omasum), no degradation could be shown *in vitro* with inoculum from the abomasum [68,100,101].

Most studies measuring OTA disappearance from the rumen *in vivo* (in this case a combination of degradation by the microbial population, passage from the rumen and potentially some association with particulate fractions) arrive at half lives of 0.6–3.8 h (average 2.8 ± 1.5 h), the OTA concentration being completely back to 0 after 6–13 h (Table 2). The range of OTA doses applied is considerable: From 9.5 μg/kg bw [11] to 500 μg/kg bw [72]. As can be expected, *in vitro* studies (disappearance of OTA due to microbial degradation and potentially some association to particulate fractions) arrive on slightly higher values for half life of 0.2–12.7 h (average 3.6 ± 3.3 h), in most studies OTA concentrations being back to zero after 6–32 h (Table 3).

**Table 2:** Ruminal disappearance of ochratoxin A (OTA).

OTA dose [μg/kg bw]	Diet	Ruminal OTA disappearance parameters	Ref.
500	100% forage (hay)	half life 0.65 h, back to zero after app. 6 h	[72]
500	100% concentrate	half life 1.30 h (30% intake), 3.38 h (100% intake); not back to zero after app. 10 h	[72]
500	100% forage (hay)	half life 0.63 h, back to zero after app. 6 h	[73]
500	100% concentrate	half life 2.67 h, not back to zero after 12 h	[73]
9.5, 19.0 and 28.5	70% concentrate	half lives 2.60, 3.76 and 3.82 h, back to zero after app. 10–13 h	[11]
14.3	70% roughage	back to zero after app. 6 h	[104]
14.3	70% concentrate	back to zero after app. 13 h	[104]
27.6	70% concentrate	half life 4.1–5.1 h; back to zero between 10 and 24 h	[105]

**Table 3:** Investigations on ochratoxin A (OTA) degradation during *in vitro* fermentation.

OTA in rumen fluid [mg/L]	Diet of donor animals	*In vitro* treatment	OTA degradation parameters	Ref.
~0.5	rumen fluid from slaughterhouse	-	After 15 min 50% degraded; after 4 h only 5% left	[68]
0.24–4.6	not given	-	0.06–0.52 mg/(h*L)	[99]
2.5	100% hay	-	0.345 mg/(h*L)	[72]
2.5	100% conc.	-	0.073 mg/(h*L)	[72]
0.2	100% hay	-	Half life 12.7 h; reduced, but not back to zero at 48 h	[107]
0.2	80% hay	-	Half life 4.1 h; back to zero at app. 24 h	[107]
0.2	50% hay	-	Half life 5.7 h; back to zero at app. 24 h	[107]
0.2	40% hay	-	Half life 3.9 h; back to zero at app. 24 h	[107]
0.2	40% hay	-	Half life 3.4 h; back to zero at app. 12 h	[107]
0.2	40% hay	+ starch	Half life 2.0 h; back to zero at app. 32 h	[107]
0.2	7–8 kg DM hay, 5–6.6 kg DM conc.	-	Half life 0.88 h; k = 0.34 h^-1^; back to zero at app. 6 h	[100]
0.2	7 kg DM hay, 4 kg DM conc.	-	Half life 1.33 h; k = 0.23 h^-1^; back to zero at app. 6.5 h	[100]
0.2	Grass ad libitum, 3 kg DM conc.	-	Half life 0.17 h; k = 1.75 h^-1^; back to zero at app. 1.5 h	[100]
0.2	Grass ad libitum, 2 kg DM conc.	-	Half life 0.51 h; k = 0.58 h^-1^; back to zero at app. 4 h	[100]
0.2	72% grass/18% grass hay, 10% conc.	-	k = 0.38 h^-1 ^± 0.13	[108]
0.2	32% grass/18% grass hay, 50% conc.	-	k = 0.49 h^-1 ^± 0.07	[108]
0.2	90% grass silage, 10% conc.	-	k = 0.21 h^-1 ^± 0.06	[108]
0.2	50% grass silage, 50% conc.	-	k = 0.29 h^-1 ^± 0.16	[108]
0.2	90% grass hay, 10% conc.	-	k = 0.22 h^-1^± 0.07	[108]
0.2	50% grass hay, 50% conc.	-	k = 0.38 h^-1 ^± 0.15	[108]
0.2	40% hay, 60% conc.	-	Half life 3.7 h; back to zero at app. 32 h	[106]
0.2	100% hay	-	Half life 4.5 h; back to zero at app. 32 h	[106]
0.2	not given	+ starch	Half life 1.9 h; back to zero at app. 32 h	[106]
0.2	Diet with monensin	-	Half life 20.1 h; not back to zero after 32 h	[106]
0.07	not given	-	Half life 3.23 h (wheat OTA); back to almost zero at 12 h; Half life 3.06 h (crystalline OTA); back to zero at 12 h	[109]

conc. = concentrate.

Non-toxic OTα is not further metabolized and would accumulate in a rumen-like environment [101,102]. On individual occasions, ochratoxin C (OTC; difference to OTA: ethyl-esterified carboxy-group of phenylalanine) [69] or other, unknown substances [103] have been described as metabolites, but at least in the former case a confusion of a protein-OTA complex with OTC can not be excluded completely [101]. *In vitro* results also indicate that any OTC appearing in the rumen would be metabolized to OTA at a high rate, and therefore finally be degraded to OTα [101].

According to several studies, OTA degradation can be considered a first-order reaction, which means that the reaction is dependant on the concentration of the substrate mainly. No blocking effect of developing OTα has been shown. The existence of first-order kinetics is explained by the fact that OTA has to pass into the cell to be degraded, and that any such concentration-dependant influx can be considered a first-order process [100]. It has been suggested that little extracellular proteolytic activity contributes to OTA degradation. This is supported by the lack of comprehensive OTA degradation activity in the fluid phase of inoculum of *in vitro* studies investigating the different rumen microbial groups concerning their OTA degrading capacity [69,72,99]. The fact that OTA follows monoexponential decay also implies that some low concentration of OTA will stay in the rumen fluid longer than what might be expected from half life alone.

#### 3.3.2. OTA degradation capacity of different microbial groups

Different groups of microbes (protozoa, bacteria or fungi) could be considered to contribute in metabolising OTA. The predominant microorganisms in the rumen are generally capable of utilizing a variety of substances, however there are contradictory studies that showed that rumen microflora is very limited and specific in toxin degradation capacity and activity and requires specific substrate for growth and as such fill a unique ecological niche. 

Concerning OTA degrading capacity of different rumen microbes (Table 4), from the beginning protozoa were found to be the most active microbes in OTA degradation [69], as confirmed repeatedly [72,99,106].

**Table 4:** Ochratoxin A (OTA) degradation by different fractions of rumen microbes.

OTA in rumen fluid	Microbial fraction	Degradation rate OTA	Ref.
~12.5 mg/L	Protozoa^1^	54% degraded after 24 h	[69]
~12.5 mg/L	Heavy bacteria^1^	13% degraded after 24 h	[69]
~12.5 mg/L	Light bacteria^1^	No degradation after 24 h	[69]
0.2 mg/L	Protozoa + heavy bacteria (200 g; 10 min)	app. 90% degraded after 4 h	[99]
0.2 mg/L	Bacteria (supernatant)	app. 10% degraded after 4 h	[99]
0.2 mg/L	Rumen fluid minus protozoa^2^	app. 15% degraded after 4 h	[99]
1250 mg/L	particulate fraction (centrifugation 10 min at 150 g)	201 μg/(h*L) (hay diet)	[72]
1250 mg/L	supernatant centrifugation	17 μg/(h*L) (hay diet)	[72]
0.2 mg/L	Protozoa fraction	Half life 2.44 h; back to zero at app. 32 h	[106]
0.2 mg/L	Bacteria fraction	Half life 99.4 h; not back to zero after 32 h	[106]
Not given	Rumen bacteria	Able to degrade OTA	[98]

^1 ^Fractionated centrifugation for 5 min at 166 g for protozoa, for 10 min at 1,500 g for heavy bacteria and for 20 min at 20,000 g for light bacteria. ^2 ^Treated with dioctyl sodium sulfosuccinate.

The overall capacity of rumen fluid to degrade OTA was shown to decrease if rumen fluid is collected shortly after feeding [99] and is related to the variation in the composition of protozoa due to change in dietary composition and feeding time. However, investigations on the contribution of different groups of protozoa (*Entodiniinae, Diploiniinae, Ophryoscolecinae and Isotrichidae*) gave no indication for clear differences between the OTA degradation capacities of these groups [101]. 

Some information indicates that gut bacteria can also have considerable OTA degrading capacity. Degradation of OTA by microbes of the hindgut (large intestine/caecum) of rats, pigs and humans (all known to lack a protozoal population) shows that gut bacteria are also capable of OTA degradation. Results of some studies indicated significant capacity for OTA degradation of the rumen bacterial fraction, too [98,99].

The role of fungi has not been investigated comprehensively. When considering their high proteolytic capacity [110], and the fact that cellulose increased OTA degradation [102], fungal OTA degradation activity in the rumen could be present [102].

#### 3.3.3. Influence of diet composition on OTA degradation

Any influence of diet composition on ruminal OTA degradation will most likely be mediated by its influence on the microbial population. Given their prominent role, influence on protozoa is most interesting. Protozoa populations can be influenced by dietary factors like diet composition, feeding level and frequency of feeding, mediating important variables of the rumen habitat like available substrates, pH or turnover rate [111]. Obvious interrelations between these variables exist and complicate simple conclusions: High concentrate diets will lower pH much less when being fed at low feeding level only, and any lack of particles to attach may have less consequences for protozoa when overall turnover rate of the rumen is low.

It appears that diets with 40–60% concentrate promote the highest protozoa density [112]. In diets of low concentrate level energy limits the population at some point. In diets with a high concentrate level low pH is likely to occur due to fast short chain fatty acid production and little stimulation of chewing and therefore limited buffer influx *via* salivation. The pH in the rumen can vary from more than 7.0 on a roughage diet to less than 5.0 on high grain diets [113,114,115], the effect of concentrate on the pH being dependant of the feeding level and adaptation of the animal to the diet. When dairy cows are consuming a total mixed ration (TMR) twice a day, the ruminal pH may range from 5.5 to 6.5 and on fresh high quality pasture, the pH may range from 5.6 to 6.8 within a 24 hour period [116]. Ruminal pH generally continues to decline 4–6 hours after feeding [117,118]. Total duration of pH drop below certain thresholds are considered to be most significant for the effects of pH changes [119]. Since protozoa limit being washed out from the rumen actively by attaching to larger particles, a reduction of such structures to attach and therefore to limit outflow may be a constraint for the protozoa population, too. The percentage of *Entodinium* usually increases as the amount of concentrate in the diet increases, and most of the time results in an *Entodininium*-only fauna or complete disappearance of the protozoa.

Diet influences OTA degradation primarily *via* its influence on the composition of the rumen microbial population. In fact, when comparing a diet with an extreme level of concentrate of 100% with a forage-only diet, much higher microbial OTA degradation activity in the rumen of hay fed sheep was found [72]. For less extreme diets (70% concentrate and 30% forage and vice versa), an *in vivo* study in sheep also found slower OTA degradation in the high-concentrate diet indicating the importance of concentrate to forage ratio in this respect [104]. *In vitro* studies are supporting this view: In accordance with the idea of maximal protozoa populations at intermediate concentrate levels, a higher OTA degradation rate in a diet consisting of 40% roughage and 60% concentrate compared to a diet consisting of 100% hay was found [107]. By adding starch, ruminal OTA halftime was 1.9 h while it increased to 4.5 h by the addition of cellulose [106]. Also in another study a higher proportion of concentrate (50% *vs.* 10% DM) resulted in a higher degradation of OTA [108].

Interestingly it was shown *in vitro* that diets high in fresh grass improved OTA degradation compared to grass hay or grass silage based diets [100,108]. Capacity of forages to promote OTA degradation was ranked to be grass > grass hay > grass silage [102]. It is not clear whether this is due to a more prominent proteolytic population (as indicated above), or due to a generally higher microbial density on the grass based diets. 

#### 3.3.4. Further aspects of rumen microbial degradation of OTA

Some influence of dosing in relation to feeding time has been reported by two independent studies, consistently finding lowest activity directly after feeding, and higher activity before and 4–6 h after feeding [9,72]. While fluctuations in the microbial population have been proposed as an explanation, the long generation time of protozoa (6–15 h for *Entodinia*, and 24–48 h for other groups; [120]) makes them rather unlikely to undergo population changes in such short time frames. At least contributing to a decrease in activity is the dilution of protozoa concentration due to an increased flux of water into the rumen after a meal, which is caused by increased drinking and salivary activity and osmolytically driven water-flux into the rumen *via* the rumen wall. Some competition of OTA with postprandially abundant food protein for proteolytic enzyme capacity should also be considered to contribute to this effect. As could be expected from investigations on other catabolic microbial activity, rumen fluid of cattle and sheep did not show a difference in OTA degradation [99].

Concerning any interpretation of OTA disappearance from the rumen fluid it must also not be forgotten that some association of OTA with all kinds of particles (e.g., bacteria, fungi and food particles) can occur, which may also detract OTA from the fluid phase to some extent (being still available for the host animal). As mentioned, degradation measured *in vitro* (non-continuous culture!) contrasts slightly from disappearance *in vivo*, since in the latter situation, rumen passage will contribute to OTA turnover depending on the level of feed intake. Based on fluid passage rates from the rumen, a rumen bypass of 2 up to 6.5% of ingested OTA for sheep with maintenance requirements was estimated for the first hour of incubation (with a maximum of 10% after 4 h) [102]. Higher feeding levels increase ruminal passage rate, thereby increasing ruminal bypass proportion. 

An influence on digestibility as stated in some studies [11] may be best explained to be a result of the diarrhea induced by the mycotoxin and not by significant direct negative effects on microbes. However, other studies found no (*in vivo*, [10]) or only slight indications (*in vitro*, [121]) for a negative influence of OTA on digestibility. Using four toxin concentrations of 5.0, 20.0, 200 and 1,400 μg/L of incubation medium, a significant decrease of *in vitro* digestibility was found only at the highest concentration [122].

## 4. Systemic Occurrence and Excretion of OTA in Ruminants

### 4.1. OTA in pre-ruminating ruminants

Compared to the plethora of reports on diverse OTA-caused pathological lesions in monogastric animals such as pigs [1,29,123,124] or poultry [3,5,125], there is comparatively little evidence and reports on pathological effects of OTA in ruminants. Acute toxicity can be demonstrated for young ruminants (functional monogastrics) without developed rumen and therefore lacking a functional microbial population in their guts (Table 5). Calves died within 24 h from single OTA doses of 11,000 and 25,000 μg/kg bw (n = 2, 5 weeks old) [126] or from a dose of 4,000 μg/kg bw (n = 2, 3 weeks old) [9]. At lower doses (1,000 and 500 μg/kg bw; n = 2 each), one of two or no calves died in the latter study.

Calves even survived a 30 d OTA dosage of up to 2,000 μg/kg bw per day. However they showed some serious signs of intoxification (polyuria and general depression, plus some kidney degeneration even at the lowest dose of 100 μg/kg bw) [127]. Since the calves used in this study were already 2–3 month old and received a pelleted diet 1–2 month before the start of the trial, some development of rumen flora may already have started, and further development due to the start of hay feeding at the same time as OTA dosing reduced negative effects of OTA on these animals. In fact, the authors explain the improvement of the situation during the 30 day dosage period by the increased detoxification capacity of the developing rumen microbial population.

### 4.2. OTA in functional ruminants

The fact that even rather young animals with developed rumen have been shown to be much less affected by OTA than pre-ruminant calves [9] indicates the significance of ruminal degradation of OTA for detoxification. This is underlined by results of intravenous dosing of OTA (Table 6). As can be expected, such total circumvention of the rumen results in significant toxicity in adult ruminants, up to death of sheep at a single dose of 1,000 µg/kg bw [128], which can be considered much less critical when given orally [126]. However, sheep appeared to be relatively little affected by a single intravenous dose of 200 µg/kg bw [73].

There are some old anecdotal and sometimes speculative reports on potential harmful effects of dietary OTA on adult ruminants from agricultural practice. An example are reports on the potential occurrence of abortions in dairy cattle after consumption of mouldy lucerne/grass forage [129,130]. The death of 15 of 30 cows due to ochratoxicosis when these animals were fed an improperly ensiled oats-lucerne mixture was reported [131]. Negative consequences ranging from general depression, fever diarrhoea and uraemia until death (113 out of 11,500 animals) in connection with affection of kidneys in steers, cows and ruminating calves after consuming OTA-contaminated diets were mentioned on another occasion [132,133], both as cited in [59].

From the background of these reports, a further look on animals with a functional rumen seems warranted irrespective of the evidence of comprehensive OTA degradation in the rumen. In a study focusing on OTA analytical methods, losses of cattle after the consumption of mouldy wheat contaminated with OTA at a level of 100 µg/kg were mentioned [134], implying a potential influence of OTA. It has to be added here that in studies reporting on farm outbreaks of mycotoxin intoxication, pathological effects can well be due to other mycotoxins like citrinin, as has been explicitly stated by some authors [133,134]. OTA is often associated with other mycotoxins like citrinin, penicillic acid or hydroaspergillic acid [126], contributing significantly or even dominantly to effects on animals and microbes if present in sufficient amounts. This factor also needs to be taken into account when using naturally OTA contaminated feeds in more defined trials.

**Table 5:** Effects and systemic occurrence of ochratoxin A (OTA) in preruminant calves.

OTA dose [µg/(kg bw*d)]	Dosing method	Duration	Animal age	n	Clinical effects; systemic presence of OTA + OTα	Excretion of OTA + OTα in urine and faeces	Detection limit chemi-cal analysis	Diet	Ref.
500	ST	Single dose	16–21 d (60 kg)	2	Both calves survived; no suppressive effect on feeding; serum contents app 0.2–0.4 µg OTA/mL (still 5 d after dosing)	Dose recovery 97%; 88% as OTα and 3.4% as OTA in urine; 9.2% as OTA in faeces	50 ng/mL HPLC	Milk	[9]
1000	ST	Single dose	10–15 d	2	1 calf dead within 12 h, 1 calf survived; labored beathing, severe diarrhea, prostration; cessation of feeding for 4 h in the surviving calf	-	-	Milk	[9]
4000	ST	Single dose	10–15 d	2	Dead within 24 h; labored beathing, severe diarrhea, prostration; refused to feed for 4 h	-	-	Milk	[9]
11000	ST	Single dose	35 d	1	Dead within 24 h (epicardial hemorrhages)	-	-	Not given	[126]
25000	ST	Single dose	35 d	1	Dead within 24 h (epicardial hemorrhages)	-	-	Not given	[126]
100, 500	CA	30 days	2 month	1+1	Polyuria on app. day 20; tended to revert to normal at the end of experiment; necropsy: pale kidney, mild enteritis, mild tubular kidney degeneration	-	-	Start of roughage feeding at start experimental period	[127]
1000, 2000	CA	30 days	2 month	1+1	Polyuria, depressed (on day 14 in the low and day 2 in the high dose), dehydrated; symptoms tended to revert to normal at the end of experiment); necropsy: pale kidney, mild enteritis, mild tubular kidney degeneration	-	-	Shift milk replacer to pellets first month of age; start roughage feeding at start experimental period	[127]

n = sample size; ST = *via* stomach tube; CA = orally in capsule; µg/(kg bw*d) = µg OTA per kg body weight and per day.

**Table 6:** Effects and systemic occurrence of ochratoxin A (OTA) when applied intravenously (single dose).

OTA dose [µg/(kg bw*d)]	Animal, age, bw	n	Clinical effects; systemic presence of OTA + OTα	Excretion of OTA + OTα in urine and faeces	Detection limit/chemical analysis	Diet	Ref.
200	Sheep, adult, 50 kg	4	Seemed normal; urine volume increased; maximal 4 µg OTA/mL in blood serum	Dose recovery (6 d) 57–61%; excretion in urine 93% as OTA and 3.2% as OTα, in faeces 4.4% as OTα	50 ng/mL, HPLC	Hay	[73]
250	Cattle, not lactating or pregnant, 400 kg	1	Not mentioned	Only OTA in urine, no OTα	TLC	Dairy ration	[130]
250	Calves, 19–20 d, 44 kg	2	1 dead after 31 h; 1 survived; no cessation of feed intake; serum OTA from 3.0 to 0.1 µg OTA/mL during 5 d	Dose recovery 70%; excretion in urine 36% as OTA; in faeces 64% as OTA; no OTα in urine or faeces	50 ng/mL, HPLC	milk at 10% bw (over night fast)	[9]
1000	Sheep, 135days pregnant	2	Dead after 12 and 24 h; pulmonary congestion and edema; liver necrosis; serum OTA from 7–8 to 1–3 µg OTA/mL during 12 h	-	not given	not given	[128]

n = sample size; TLC = thin layer chromatography; HPLC = high pressure liquid chromatography; n.g. = not given.

Obviously only studies dosing OTA orally represent the situation of OTA in functional ruminants in a realistic way. In this respect it can be considered rather irrelevant whether the dosage occurred orally (deliberate intake or *via* oro-ruminal probe) or intraruminally (*via* a rumen fistula) and at least secondary whether OTA was included in the normal feed or as capsule and whether the crystalline substance or naturally contaminated feeds were used [101,109]. As a rough estimate of the OTA contamination a ruminant may have to face in a practical situation, daily doses of a size of approximately 2.0 μg/kg bw (assumptions: 600 kg bw, 20 kg DM intake; OTA contamination of diet 50 μg/kg DM) appear possible (although representing an over- rather than an underestimation of the average situation) while a daily dose of 20 μg/kg bw rather represents the maximal imaginable dose if an extreme situation is assumed (500 μg OTA/kg DM). The studies summarized in Table 7 (application of OTA >14 days, starting at a dose of 9.5 μg/kg bw) represent the practical situation much more than studies dosing OTA for a short period but at a rather high dose (Table 8; application of OTA from 1–6 days).

#### 4.2.1. Pathological findings and systemic occurrence

The lack of a general protocol (e.g., whether signs were actually searched for, or only noticed by chance when very clear) complicate comparison of studies, but conclusions can still be drawn from the information available to date. Clear signs of discomfort and starting intoxification (polyuria, reduced feed intake) are first reported at repeated doses of 225 μg/kg bw [10] or at a single dose of 500 μg/kg bw (polyuria; [73]) for sheep. At a single, but very high dose of 13,300 μg/kg bw a cow showed severe clinical signs like difficulties in arising, diarrhoea, anorexia and cessation of milk production, the latter only recovering to one third of normal during this lactation [126]. In the same study, an adult goat died within 5–6 days after daily oral OTA doses of 3,000 μg/kg bw. This is not to say that such effects can not occur at lower levels but rather that one can expect such effects at the OTA doses used in these studies.

Little work has been published on changes of the target tissues of OTA like kidney or liver. While only minimal microscopic kidney changes were reported in goats fed a high OTA dose for 14 days [126], another study did not find pathological kidney changes at practically relevant OTA levels and dosage periods [70]. 

While such information could be interpreted as a justification of considering OTA as a mycotoxin of no relevance and completely uncritical in ruminants, repeated reports on the systemic occurrence (like in blood and in urine) not only of the harmless OTα metabolite, but also of OTA show that detoxification is less than might have been expected. Even repeated doses of 9.5 μg/kg bw [11] led to OTA in blood serum and urine of sheep. Overall, transfer of OTA into blood is linearly increasing with the dose of OTA [11]. 

**Table 7:** Systemic occurrence of ochratoxin A (OTA) and ochratoxin α (OTα) in functional ruminants after oral dosing over >14 days.

OTA dose [µg/(kg bw*d)]	Study duration	Animal, age, bw	n	Clinical and pathological effects; systemic presence of OTA + OTα	Excretion of OTA + OTα in faeces and urine	Detection limit/ chemical analysis	Diet	Ref.
9.5	29 d	Sheep, 1 year, 39 kg	3	No overt illness; food intake not influenced; blood serum OTA 1.5–6.0 ng/mL, OTα 0.4–0.8 ng/mL	Dose recovery 80% (7 d); 1.9% OTA and 20.4% OTα in faeces, 7.8% OTA and 70% OTα in urine	0.2 ng/mL (HPLC)	70% conc. + 30% grass silage	[11]
14	31 d	Sheep, adult, 58 kg	3	No overt illness; food intake not influenced; blood serum OTA 2–4 ng/mL	Dose recovery 81% (7 d); 1.5% OTA and 11.2% OTα in faeces, 4.4% OTA and 82.9% OTα in urine	0.2 ng/mL (HPLC)	70% roughage	[104]
14	31 d	Sheep, adult, 58 kg	3	No overt illness; food intake not influenced; blood serum OTA 4–9 ng/mL (tendency to accumulate).	Dose recovery 78% (7 d); 0.9% OTA and 18.5% OTα in faeces, 5.8% OTA and 75% OTα in urine.	0.2 ng/mL (HPLC)	70% conc.	[104]
14.7–16.5	87 d	Cattle, 12 weeks, 80 kg	6	No liver, kidney, skeletal or heart muscle damage; 3 calves with some OTA in kidney	no OTA in urine but some OTα	-	1.5 kg hay + 1.5–2.7 kg conc.	[70]
12.0–16.0^1^	87 d	Cattle, 12 weeks, 80 kg	6	No liver, kidney, skeletal or heart muscle damage; 2 calves with some OTA in kidney	no OTA in urine but some OTα	-	1.5 kg hay + 1.5- 2.7 kg conc.	[70]
~18	77 d	Cattle, adult, app. 400 kg	2	Clinically normal; lesions on kidneys, subacute interstitial nephritis; some OTA detected in kidneys of one animal; no OTA or OTα in milk, muscle or liver reported	No OTA or OTα detected in urine.	-	9 kg hay + 10 kg conc.	[135]
19	29 d	Sheep, 1 year, 39 kg	3	No overt illness, food intake not influenced; blood serum OTA 4.6–12.4 ng/mL, OTα 0.7–2.3 ng/mL	Dose recovery 78% (7 d); OTA 7.7% in urine and 1.9% in faeces; OTα 20.7% in faeces and 70% in urine	0.2 ng/mL (HPLC)	70% conc.+ 30% grass silage	[11]
22	28 d	Sheep, adult, 66 kg	4	No overt illness; food intake not influenced; normal weight gain; blood serum OTA 8.2–10.8 ng/mL, OTα 2.0–3.4 ng/mL	Dose recovery 75%; OTA 5.1% in urine and 1.1% in faeces; OTα 13% in faeces and 81% in urine	HPLC	70% conc.+ 30% hay	[10]
28.5	29 d	Sheep, 1 year, 39 kg	3	No overt illness; food intake not influenced; blood serum OTA 6.4–18.2 ng/mL, OTα 0.7–1.6 ng/mL	Dose recovery 74% (7 d); OTA 12% in urine and 3.4% in faeces; OTα 36% in faeces and 49% in urine	0.2 ng/mL (HPLC)	70% conc. + 30% grass silage	[11]
55	28 d	Sheep, adult, 66 kg	4	No overt illness; food intake not influenced; normal weight gain; blood serum OTA 67.0–111.7 ng/mL, OTα 12.0–18.5 ng/mL	Dose recovery 84%; OTA 4.8% in urine and 0.59% in faeces; OTα 16% in faeces and 91% in urine	HPLC	70% conc. + 30% hay	[10]
225	14 d	Sheep, adult	n.g.	Reduction in feed intake (toxicosis); blood serum OTA 36 ng/mL, OTα 15 ng/mL	-	HPLC	70% conc. + 30% hay	[10]
1000, 2000	14 d	Goat, adult, 59 kg	1,1	No clinical signs besides diarrhoea and polyuria promoting haemoconcentration (urea N up, minimal microscopic kidney changes)	-	-	Lucerne hay + conc.	[126]

n = sample size; n.g. = not given; conc. = concentrate; HPLC = high pressure liquid chromatography; ^1 ^plus aflatoxin.

**Table 8:** Systemic occurrence of ochratoxin A (OTA) and ochratoxin α (OTα) in functional ruminants after oral dosing ≤6 days.

Dose [µg/(kg bw*d)]	OTA appl.	Duration	Animal, age, bw	n	Clinical and pathological effects; systemic presence of OTA + OTα	Excretion of OTA+OTα in faeces and urine	Detection limit/method	Diet	Ref.
22 (4 d) + 55 (2 d)	FE	4+2 d	Sheep, adult, 50 kg	1	Not commented on; no OTA and OTα in serum 1 h after dose	-	n.g.	Not given	[99]
27.6	FE	Single dose	Sheep, adult, 89 kg	6	No overt illness; blood serum OTA max 14.4 ng/mL	Dose recovery 86%; OTA 6.5% in urine and 3.7% in faeces; OTα 34% in faeces and 56% in urine	0.2 ng/mL HPLC	70% conc.+ 30% grass silage	[105]
200	FE	4 d	Cattle, lactating, not pregnant, 500 kg	1	No overt clinical signs; delivery of normal calves; no OTA and OTα up to 200 μg/kg DM in milk (back to zero 1.5 d after last dose)	No OTA and up to 8 μg/mL OTα in urine	TLC	Dairy cattle ration	[126,130]
250	ST	Single dose	Cattle, not pregnant or lactating, 400 kg	1	No overt clinical signs	No OTA and up to 2 μg/mL OTα in urine	TLC	Dairy cattle ration	[130]
500	CA	Single dose	Goat, adult, 45 kg	2	Not commented on; 6% in milk and 2% in serum (in the latter 3 dominantly as undetermined metabolites)	Excretion of OTA dose: >90% within 7 days, excretion 54% in faeces (dominantly as OTA), 38% in urine	TLC	Hay	[103]
500	CA	Single dose	Goat, adult, 45 kg	2	Not commented on; 1.5% and 0.5% of total dose found in liver and kidney 6 h after dose	-	TLC	Hay	[103]
500	RC	Single dose	Sheep, adult, 60 kg	2	No overt illness; notion of increased urine volume; in blood serum OTA up to 400 ng/mL at 100% intake and 150 ng/mL at 30% intake 4 h after dose	Dose recovery 67%; OTA 1.2–2.8% in urine and 0.28–0.29% in faeces; OTα 7.6–18% in faeces and 81–89% in urine	HPLC	100% grain	[73]
500	RC	Single dose	Sheep, adult, 60 kg	2	No overt illness; notion of increased urine volume; in blood serum OTA up to 100 ng/mL 4 h after dose	Dose recovery 58%; OTA 0.56% in urine and 0.93% in faeces; OTα 24% in faeces and 75% in urine	HPLC	100% hay	[73]
500	RC	Single dose	Sheep, adult, 20 kg	4	No overt illness; area-under-the-curve (AUC) blood serum OTA 6495 (ng*h/mL) and OTα 196 (ng*h/mL)	-	HPLC	100% grain	[73]
500	RC	Single dose	Sheep, adult, 20 kg	4	No overt illness; area-under-the-curve (AUC) blood serum OTA 1456 (ng*h/mL) and OTα 494 (ng*h/mL)	-	HPLC	100% hay	[73]
750	ST	5 d	Cattle, 3 mon pregnant, not lactating, 600 kg	1	Delivery of normal calves; in milk no OTA, but traces of OTα	Traces of OTα in urine	TLC	Dairy cattle ration	[126,130]
1660	ST	5 d	Cattle, 6 mon pregnant, lactating, 600 kg	1	Delivery of normal calves; in milk OTA app. 100 μg/kg DM on day 3,4 and 5; back to zero 2 days after dose; OTα 750 μg/kg DM on day 1–6	Traces of OTα in urine	TLC	Dairy cattle ration	[126,130]
2000	ST	Single dose	Cattle, 46–69 d, 68–100 kg	4	No overt illness; in blood serum OTA 2.0–0.1 ng/mL (decrease over 5 d) and OTα 0.1–0.2 ng/mL	Dose recovery 92%; excretion as OTA 0.4% in urine and 1.9% in faeces; as OTα 82% in urine and 16% in faeces	50 ng/mL	Barley + hay	[9]
3000	ST	5 d	Goat, adult, pregnant, 59 kg	1	Dead after 5 days; diarrhea, dehydration; no gross lesions; microscopical lesions confined to centrolobular swelling of liver	-	-	Lucerne hay + conc.	[126]
13300	ST	Single dose	Cattle, lactating, 6 months pregnant, 600 kg	1	Difficulty in arising, diarrhea, anorexia 1–4 d after dosing; drastic reduction of milk production; in milk OTA up to 640 μg/kg DM, OTα 4500 μg/kg DM after one day	Traces of OTα in urine	TLC	Dairy cattle ration	[126,130]

FE = orally in feed; ST = *via* stomach tube; RC = rumen cannula, CA = orally in capsule; appl. = application; HPLC = high pressure liquid chromatography; TLC = thin layer chromatography.

#### 4.2.2. Excretion *via* urine and faeces

Apparently, intravenous dosage of OTA seems to circumvent all sites of degradation of OTA to OTα, since in two studies, 90–100% of the dose was found to be excreted as OTA [73,130]. It can be safely concluded that OTA degradation is very limited after absorption from the gut. The main route of excretion of OTA in functional ruminants dosed orally is as its metabolite OTα *via* urine. Interestingly, in a study using young ruminants most of the OTA dose was found to be excreted in the form of OTα [9]. This could stem from degradation of OTA by animal or microbial enzymes. Potentially, the calves investigated already had some amylolytic population, which would also develop significant proteolytic activity, even if there is not yet significant fibrolytic activity present. 

#### 4.2.3. Particularities influencing OTA toxicity and degradation in ruminants

The systemic occurrence of OTA in ruminants despite the microbial population actively degrading the toxin can best be explained by the fact that the rumen functions comparable to a mixing chamber, which allows some outflow (bypass) of any substrate entering the rumen [11], irrespective of the measured long mean retention times. OTA escaping ruminal degradation will readily be absorbed in the lower digestive tract, just as in non-ruminants.

Several factors have been discussed in their influence on OTA toxicity in ruminants, most of them connected to ruminal degradation. A significant influence of diet (mostly quantified as concentrate to roughage ratio) has been indicated by two studies [73,104], pointing to a more comprehensive bioavailability of OTA in concentrate based diets. Besides influencing the microbial population (as outlined above in more detail), a low rumen pH will also increase the occurrence of OTA in its non-dissociated (“protonated”) form, which will also facilitate absorption of OTA from the rumen and therefore systemic availability of the toxin [11]. Based on the consideration that general proteolytic enzymes catalyze the cleavage responsible for OTA detoxification, a signifcant improvement of degradation capacity *via* adaptation of the microbial population does not seem probable, e.g., in contrast to fibre digestion.

There are further relevant aspects for systemic OTA metabolism in ruminants. Biliary secretion (like demonstrated in other taxa like rats) has been postulated for OTA [73], explaining repeated peaks in blood concentrations of OTA and its metabolites and the occurrence of OTA in the faeces of animals dosed intravenously.

The binding of OTA to blood serum proteins determines its excretion rate and therefore its toxicity. Since in cattle as in humans and pigs binding is 2–3 times stronger than in sheep, the latter appear less prone to accumulation of OTA in blood [11]. The high urinary pH of herbivores facilitates OTA clearance from blood compared to rhesus monkeys, pigs and rats [11]. In general, chronic ingestion of comparatively low levels of OTA represents the situation in practice, and it can be considered the more critical scenario, having some potential to lead to accumulation in the long run.

OTα has been shown to be almost non-harmful on a cellular level in toxicology studies [136,137]. The far weaker bondage of OTα to blood serum proteins and therefore faster excretion is generally considered to be another key feature for its much lower toxicity compared with OTA [138,139].

### 4.3. Occurrence of OTA in ruminant tissues (meat)/products

For tissue of monogastric animals, there is clear evidence for the presence of OTA in pig meat and meat products [140]. Since OTA is accumulated in kidneys, this organ is contaminated most significantly, followed by liver, muscle and fat. The incidence of OTA in different pig-based sausages ranged from 46.7 to 77.2%, with maximum OTA concentrations of 3.16 or 4.56 µg/kg blood or liver-type sausages, respectively [140]. OTA was essentially absent in meat from poultry, but present in considerable amounts in blood [141] and high concentrations in kidneys [1].

Ruminant organs or muscle meat are not considered to be contaminated by OTA [142]. Given the accumulation in the kidney in other species, this would be expected to be the most likely tissue to be OTA contaminated. Overall, OTA was only detected in five of 31 beef sausage samples. It was noted that OTA contamination of ruminant meat products may also arise from the use of pig blood plasma and OTA contaminated spices [140].

In calves fed diets containing OTA, the non-toxic cleavage product OTα has been found in trace amounts in the blood and at levels of <10 µg/kg in kidneys [70]. When two cows were fed a diet containing OTA at 317 to 1125 µg/kg of feed for 11 weeks, OTA was found in some tissues but not in milk [135]. In the later study, muscle contamination was investigated, but results not reported explicitly, indicating no presence of OTA in this tissue.

### 4.4. Occurrence and transfer of OTA into ruminant milk

In general, carry-over of OTA into ruminant milk is considered to be of little significance [6,25], especially if compared with monogastric species or other mycotoxins like aflatoxin. However, some studies found a transfer of OTA into ruminant milk, too. OTα was found in the milk of cows when being fed an OTA contaminated diet (in a single dose of 13.3 mg/kg and daily doses of 1.66, 0.75 and 0.25 mg/kg; one cow per treatment) [126]. For the cow with the very high single dose, OTA was found in milk in high amounts one day after the treatment. Traces of OTA were found on days 3, 4 and 5 after the start for the cow on the 1.66 mg/kg dose of OTA, and no OTA was found at daily doses <1.66 mg/kg. In goats given a single dose of 0.5 mg OTA/kg, recovery of OTA and OTA metabolites in milk was about 6% of the total excretion (faeces: ~54%; urine 38%; 2.3% in serum) [103]. Of this 6%, about 14% were in the form of OTA, and the rest in the form of 3 other metabolites not further characterised.

In other experimental studies no transfer of ochratoxins into milk of ruminants could be shown: Concentrations of 0.317–1.125 mg OTA/kg in the concentrate (diet as offered: 10 kg concentrate + 9 kg hay per day) did not lead to detection of OTA or OTα in the milk of cows [135]. In ewes being offered 1.5 kg hay plus 0.5 kg OTA-contaminated concentrate (OTA content in concentrate 0.04 mg/kg, approximate dose 0.2 μg/kg bw), no ochratoxin was found in milk [143] after a period of 10 days, too, just as in another study by these authors using regular sheep diets only occasionally contaminated with OTA [144].

In surveys on samples from milk producers, no OTA and no or only traces of OTα were detected in 121 samples of cow milk from northern Germany [145]. Another study also found no OTA in 69 milk samples from Germany [146]. OTA was also absent in 100 dairy milk samples of a milk survey in the UK [147]. In this study, 100 milk samples (50 retail and 50 farm gate), both produced conventionally and organically, were analysed. Spanish studies also found no OTA in 48 [148] and 12 [149] milk samples.

On the contrary, traces of OTA were found in cow milk in a Swedish study [150]. A total of 36 cow milk samples were analysed. OTA was found in five (14%) of the samples (range 0.01–0.04 ng/mL). Small traces of OTA in milk were also found in a Norwegian study [151]. Samples of cow milk from organic and conventional farms and cow milk-based infant formulas were analysed. OTA was detected in six out of 40 cow milk samples from conventional farms (range 0.011–0.058 ng/mL) and in five out of 47 organic farm milk samples (range 0.015–0.028 ng/mL). OTA was not detected in any of the 20 infant formula samples. The OTA levels in cow milk found in this investigation were sufficient to cause a higher intake of OTA than the suggested tolerable daily intake of 5 ng/kg bw, e.g. in small children who consume large quantities of milk. In a survey on raw bulk milk in the northwest of France, in 132 farms surveyed in winter and summer using diets based on maize silage and containing large portion of farm produced cereal grains, the overall incidence of milk contamination with OTA at farm level was low [152]. OTA was detected in three out of 264 samples at a low level of 0.005 to 0.008 ng/mL. OTα was not determined in this study, so it remains unclear whether the low occurrence of OTA was due to a very effective degradation of OTA in the rumen, or due to low contamination of the feeds used by the farmers. A study on five farms in the Sudan detected OTA in cattle milk in one sample at a very high concentration of 2.73 ng/mL. The animals were fed a ration containing OTA at a level of approximately 0.61 μg/kg feed [47]. In a survey on dairy products (n = 195) in Germany, low OTA incidence and concentrations were found; the most prominent contamination was found for cheese with ingredients, indicating that like in meet products, spice ingredients contribute more to OTA contamination than the animal product itself [146].

If present at all, OTA content of milk ranged from 0.005–0.058 ng/mL in different studies. Although this concentration in the milk is not to be regarded as a severe and drastic problem for the consumers it shows that limited transfer of OTA into milk can occur under certain circumstances. It needs to be added that a direct animal-independent OTA contamination of milk after the milking process can not be excluded completely.

## 5. Concluding Remarks and Considerations

Traditionally, OTA has been regarded as relatively uncritical for ruminants. Based on *in vitro* trials, 12 mg OTA/kg feed were estimated to be degradable by ruminants [68]. Another author describes repeated doses of 33–72 mg OTA/d for cattle and 3–7 mg OTA/d for sheep as being degradable by the animals [102]. This would correspond to amounts of approximately 200 μg/kg bw [68] (assuming 1.2 kg daily feed intake and 70 kg sheep) or 40–120 μg/kg bw [102] (assuming 450–600 kg cattle and 45–70 kg sheep). In fact, at estimated doses of 40–100 μg/kg bw (sheep; assuming 60 kg bw and 1.2 kg food intake) [99] could not detect any OTA in blood serum. However, it needs to be added here that in older studies, analytical limits for OTA of 75 ng/mL [99] are almost two orders of magnitude above today’s levels of app. 1 ng/mL [11]. In fact, based on improved and more sophisticated analytical methodology and equipment, some more recent evidence is available that the effect of OTA on ruminants in this respect is generally underestimated. At concentrations described to be safe, OTA occurs systemically in significant amounts [10,11,105]. Therefore, although no obvious pathological effects occur, the common view that OTA is degraded completely by an active rumen microbial population under all circumstances does not hold true. 

The most likely explanation is that rumen bypass of intact, undegraded OTA needs to be taken into account. Even when assuming an active microbial population the working modus of the ruminal forestomach always leads to some bypass of feed components, the more comprehensive the more these substances resemble fluids in their mixing and passage behaviour from the rumen. Following an approach to estimate ruminal protein degradation [153,154], assuming a best case (degradation rate in the rumen: 0.40 h^-1^; passage rate from the rumen 0.02 h^-1^) or a worst case (degradation rate in the rumen: 0.06 h^-1^; passage rate from the rumen 0.10 h^-1^) scenario results at estimates of OTA bypass of 5 up to 62 % of the dose, explaining the systemic occurrence of undegraded OTA in significant amounts irrespective of an intact and active rumen microbial population experienced by some of the more recent studies. Other authors arrive at estimates of a maximal rumen bypass of intact OTA of 10% [102].

Suitable feed additives are a future perspective to further reduce any risk of OTA in ruminants. The pH-stabilizing effects of yeasts and the potentially beneficial effect on the microbial population have already been investigated in this respect, however did not influence OTA degradation and systemic availability in the study of [105].

While several studies on OTA in ruminants have improved our knowledge significantly over the last three decades, some knowledge gaps appeared to us in the process of summing up this review. From the point of view of basic research, further systematic *in vitro* trials on the influence of diet and the relevance of different microbes on ruminal degradation of OTA appear desirable to further clarify the role of individual groups. Critical regarding the practical relevance of OTA in ruminants would be information of systemic occurrence levels and transfer into milk in ruminants fed at the high feeding levels typical for lactating animals for sufficiently long period (1–3 months) at praxis-relevant OTA-concentrations (on the lower range of those reported in Table 7). This will further assure judgements of the potential of OTA to induce harmful effects in ruminants.
